# Post-Translational Dosage Compensation Buffers Genetic Perturbations to Stoichiometry of Protein Complexes

**DOI:** 10.1371/journal.pgen.1006554

**Published:** 2017-01-25

**Authors:** Koji Ishikawa, Koji Makanae, Shintaro Iwasaki, Nicholas T. Ingolia, Hisao Moriya

**Affiliations:** 1 Graduate School of Natural Science and Technology, Okayama University, Okayama, Japan; 2 Research Core for Interdisciplinary Sciences, Okayama University, Okayama, Japan; 3 Department of Molecular and Cell Biology, Center for RNA Systems Biology, University of California, Berkeley, California, United States of Aamerica; Ohio State University, UNITED STATES

## Abstract

Understanding buffering mechanisms for various perturbations is essential for understanding robustness in cellular systems. Protein-level dosage compensation, which arises when changes in gene copy number do not translate linearly into protein level, is one mechanism for buffering against genetic perturbations. Here, we present an approach to identify genes with dosage compensation by increasing the copy number of individual genes using the genetic tug-of-war technique. Our screen of chromosome I suggests that dosage-compensated genes constitute approximately 10% of the genome and consist predominantly of subunits of multi-protein complexes. Importantly, because subunit levels are regulated in a stoichiometry-dependent manner, dosage compensation plays a crucial role in maintaining subunit stoichiometries. Indeed, we observed changes in the levels of a complex when its subunit stoichiometries were perturbed. We further analyzed compensation mechanisms using a proteasome-defective mutant as well as ribosome profiling, which provided strong evidence for compensation by ubiquitin-dependent degradation but not reduced translational efficiency. Thus, our study provides a systematic understanding of dosage compensation and highlights that this post-translational regulation is a critical aspect of robustness in cellular systems.

## Author Summary

Cells are exposed to environmental changes leading to fluctuations in biological processes. For example, changes in gene copy number are a source of such fluctuations. An increase in gene copy number generally leads to a linear increase in the amount of protein; however, a small number of genes do not show a proportional increase in protein level. We investigated how many of the genes exhibit this nonlinearity between gene copy number and protein level. Our screen of chromosome I suggests that genes with such nonlinear relationships constitute approximately 10% of the genome and consist predominantly of subunits of multi-protein complexes. Because previous studies showed that an imbalance of complex subunits is very toxic for cell growth, a function of the nonlinear relationship may be to correct the balance of complex subunits. We also investigated the underlying mechanisms of the nonlinearity by focusing on protein synthesis and degradation. Our data indicate that protein degradation, but not synthesis, is responsible for maintaining a balance of complex subunits. Thus, this study provides insight into the mechanisms for coping with the fluctuations in biological processes.

## Introduction

Robustness in biological systems is a general trait of living cells and a fundamental feature involving the maintenance of stability during perturbation [1–4]. It is a universal challenge to cope with perturbations leading to fluctuations in biological processes because cells are exposed to changes in internal and external environments [5,6]. The robustness of cells to various perturbations can be understood as a consequence of fluctuations in gene expression and buffering of fluctuations [5–8]. Therefore, understanding buffering mechanisms is essential to understanding the optimization of gene expression and adaptation to changes in environmental conditions.

The decoding of genetic information is achieved through irreversible processes from DNA to RNA to protein as stated in the central dogma of molecular biology [9]. The gene expression level at each step is generally in a linear relationship with gene copy number, namely an increase in gene copy number leads to a proportional increase in messenger RNA (mRNA) and corresponding protein levels. However, in the face of perturbations, this linear relationship should become nonlinear for maintaining cellular homeostasis. This prediction highlights the importance of studying the quantitative aspects of the central dogma in the context of robustness. For example, previous studies have investigated the robustness of gene expression level under genetic perturbations caused by an increase in gene copy number [10–12]. These efforts have demonstrated that the copy number of a subset of genes in the genome correlates with mRNA levels but not directly with protein levels. This phenomenon is known as protein-level dosage compensation, reported in yeast and mammalian cells [13–15]. Although dosage compensation is expected to contribute to cell robustness, we lack a systematic understanding of the underlying mechanisms that confer robustness to biological systems.

Systematic investigations of the robustness in cellular systems have been performed by focusing on the effects of manipulating gene copy number on cell growth [12,16–18]. We previously measured cell robustness to gene overexpression using a genetic technique termed genetic tug-of-war (gTOW), by which fragility to protein overproduction is indirectly and quantitatively assessed as an upper limit of gene copy number in *Saccharomyces cerevisiae* [17,19,20]. The genome-wide gTOW analysis has revealed fragile points as a set of 115 dosage-sensitive genes that cause impaired growth when the gene copy number is slightly increased [17]. In other words, only 2% of the yeast genome (115 out of 5806 genes) is sensitive to gene dosage such that a copy number increase leads to breakdown of biological systems. Conversely, this result indicates that genetic perturbations to biological processes are generally buffered. However, the buffering mechanisms behind the robustness against gene overexpression remain to be investigated.

In this study, we developed a screening system for genes with dosage compensation based on the gTOW technique. Here, our findings suggest that the proportion of the dosage-compensated genes in the genome is approximately 10% and that these genes may encode subunits of protein complexes. We investigated the compensation mechanism by focusing not only on protein degradation but also on translational efficiency by using a ribosome profiling technique [21]. Our data suggest that the robustness of gene expression reflects transient degradation, dynamic changes in protein lifetime, produced in response to environmental changes.

## Results

### Identification of genes with protein-level dosage compensation

**Fig 1:**
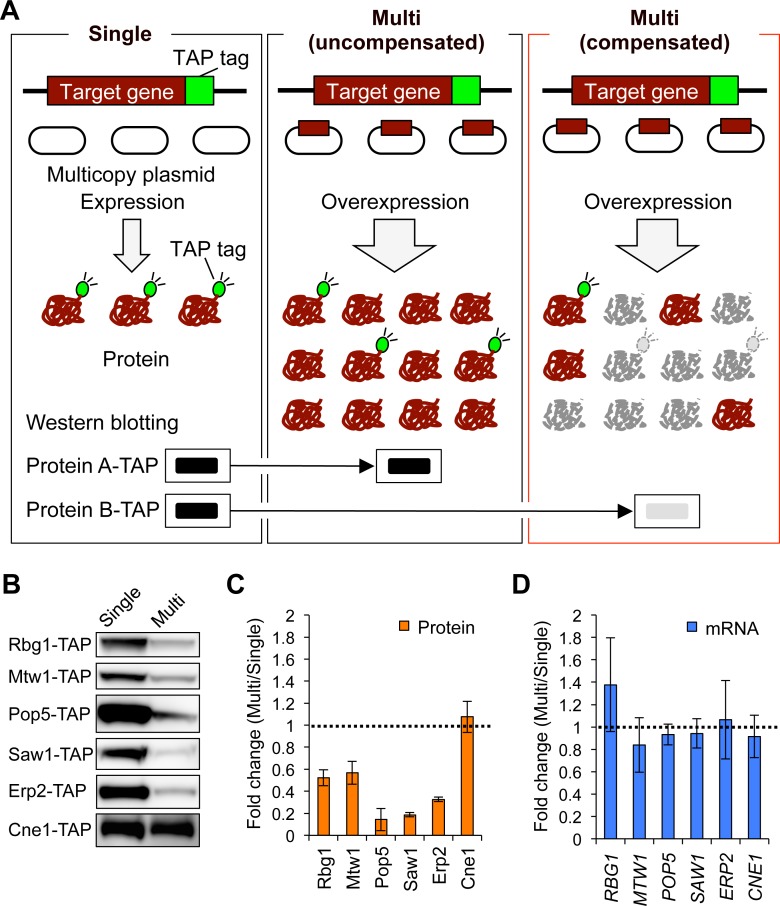
Identification of genes with dosage compensation. (**A**) Schematic overview of the screening. Left panel (Single): TAP-tagged strain transformed with the empty vector. The native level of the target protein expressed only from the genomic copy is detected by Western blotting with peroxidase anti-peroxidase (PAP). Middle and right panels (Multi): TAP-tagged strain transformed with the multicopy plasmid carrying the target gene without the TAP tag. If the level of TAP-tagged target protein is not reduced compared with that in the Single condition (middle panel), the target protein (Protein A) is not subjected to dosage compensation (uncompensated). If the level of TAP-tagged target protein is reduced compared with that in the Single condition (right panel), the target protein (Protein B) is subjected to dosage compensation (compensated). The cells carrying multicopy plasmid were grown in synthetic complete medium lacking uracil (SC–Ura). (**B**) Western blots of proteins whose expressions are reduced upon an increase in their gene copy numbers. Cne1 is an example of the uncompensated proteins. (**C, D**) Quantification of protein (**C**) and mRNA (**D**) expression levels of the identified genes. The mRNA level of each TAP-tagged target gene was measured by reverse transcriptase-PCR and normalized to *ACT1* mRNA levels. The average fold changes ± standard deviation (s.d.) from three biological replicates were calculated relative to the Single condition. Dashed line denotes the same expression level between the Multi and Single conditions.

To identify genes with dosage compensation, we developed a screening method as shown in Fig 1A. The key idea of this method is to determine the protein level expressed from a single copy of a target gene when its copy number is increased. We monitored the level of each target protein labeled with the tandem affinity purification (TAP) tag expressed from the genomic locus when the copy number of the same target gene without the TAP tag is increased by a multicopy plasmid (Fig 1A, middle and right panels). If the expression level of the TAP-tagged protein is reduced in this situation, we consider that the target gene is subjected to dosage compensation (Fig 1A, right panel), since the compensation mechanism should not distinguish the TAP-tagged endogenous protein from the non-tagged exogenous protein. Here, we call the condition where the target protein is expressed from the single genomic copy “Single” (Fig 1A, left panel) and the condition where the target protein is expressed from the genomic copy and the multicopy plasmid “Multi” (Fig 1A, middle and right panels). We used a series of strains in which the TAP tag is integrated into the 3´-region of each gene [22], and a plasmid collection in which each target gene with native regulatory regions, including promoter and 5´ and 3´ untranslated regions, is cloned into a multicopy plasmid, pTOWug2-836 [17].

We screened 54 genes on chromosome I whose TAP-tagged strains were available as representatives of the yeast genome (S1 Fig). By this screening, we identified five genes (*RBG1*, *MTW1*, *POP5*, *SAW1*, and *ERP2*) whose protein expression was reduced when their copy numbers were increased (Fig 1B). We did not detect off-target effects of an increase in gene copy number by the gTOW technique: the total cellular protein level measured in the total cell lysate did not differ in the Single and Multi conditions. An example of this observation is shown in S2A and S2C Fig. Quantification of fold change of the protein levels was carried out as shown in S2 Fig. The protein levels of the dosage-compensated genes were 0.2–0.6-fold (Fig 1C), when their copy numbers were 15–27 copies (S3 Fig). The dosage compensations are performed by post-transcriptional regulation because mRNA levels from the endogenous locus did not change even when the copy numbers were increased (Fig 1D). We thus identified five genes with dosage compensation via post-transcriptional mechanisms.

To verify the experimental setup for measuring only the endogenous protein levels, we measured the level of a target protein expressed from both the genome and plasmid. The experimental setup is the same with that used for the analysis of endogenous protein except that the plasmid encodes each of the TAP-tagged target proteins (S4A Fig). We measured the total TAP-tagged protein levels (S4B and S4C Fig) and the plasmid copy numbers (S4D Fig) and calculated the fold change of the protein levels per gene copy (S4E Fig). This analysis showed dosage compensation of all the five genes identified by the chromosome I screen when considering both endogenous and exogenous protein levels (S4F Fig). The fold change values were very similar with those calculated from the endogenous protein levels. Thus, we conclude that the experimental setup shown in Fig 1A, whereby we detect the TAP-tagged protein expressed from the genomic locus, can capture dosage compensation.

We further verified the experimental setup using green fluorescent protein (GFP) tag in order to assess the dependency of dosage compensation on the TAP tag. We used the yeast strains in which the GFP tag is integrated into the 3´-region of each target gene and measured the expression levels of GFP-tagged target proteins upon an increase in gene copy number. Western blot analysis for the dosage-compensated proteins Rbg1 and Mtw1 and the uncompensated protein Pop8 showed reduced levels of Rbg1 and Mtw1 but not Pop8 in the Multi condition (S5 Fig). Because the similar degree of the compensation was observed between the analyses using the TAP and GFP tags, dosage compensation is not a TAP-tag-mediated phenomenon.

### The ubiquitin–proteasome system is a general mechanism of dosage compensation

**Fig 2:**
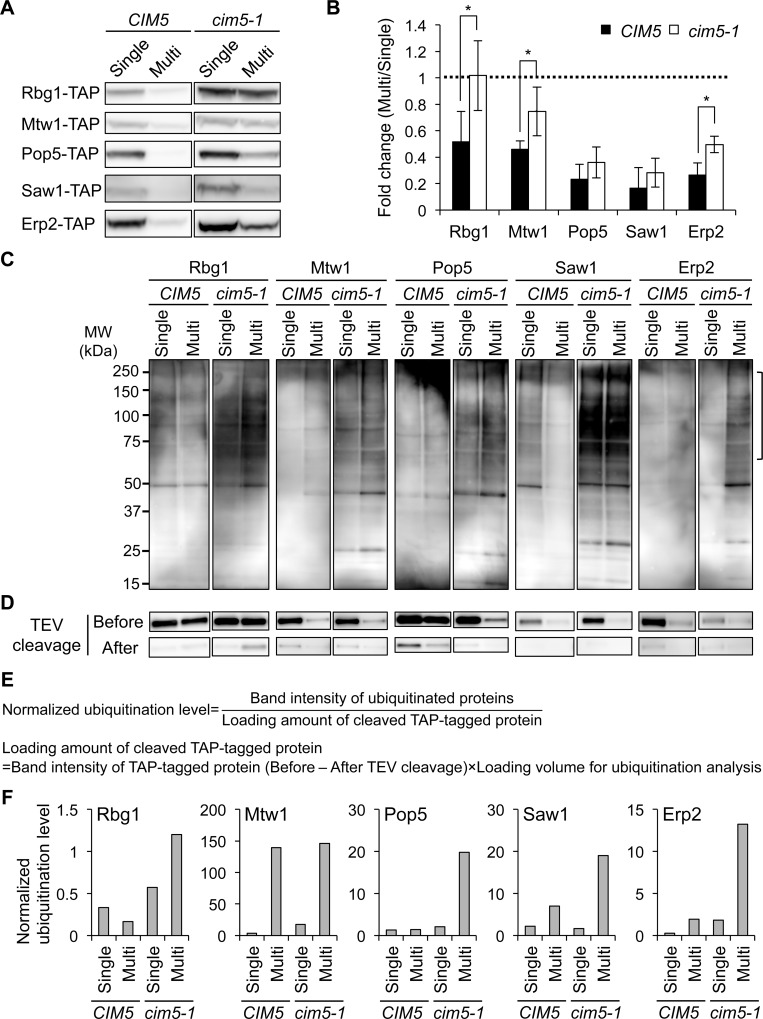
Major contribution of the ubiquitin–proteasome system to dosage compensation. (**A**) Western blots showing the amount of the indicated proteins in *CIM5* (W303-1B) and *cim5-1* (CMY765) strains grown in SC–Ura medium. The TAP-tagged target proteins expressed from the genomic regions were detected with PAP. (**B**) Quantification of the amount of the indicated proteins. The average fold changes ± s.d. from more than three biological replicates were calculated relative to the Single condition. Dashed line denotes the same expression level between the Multi and Single conditions. *P* values were determined by a one-tailed Mann Whitney *U* test (*P < 0.05). (**C**) Ubiquitination of the dosage-compensated proteins. The TAP-tagged proteins expressed in the indicated conditions were immunoprecipitated with IgG-coated beads and cleaved with TEV protease, followed by Western blotting with anti-ubiquitin antibody. The bracket indicates poly-ubiquitinated species. (**D**) Western blots of the dosage-compensated proteins captured on the beads. The TAP-tagged target proteins before and after TEV cleavage were detected with PAP. A combination of strain and condition of gene copy number in each lane is identical with Fig 2C. (**E, F**) Quantification of the levels of ubiquitinated proteins. Band intensity of ubiquitinated proteins were normalized by dividing it by loading amount of the TAP-tagged proteins (**E**). Bar graph indicates the normalized ubiquitination level of each of the dosage-compensated proteins in *CIM5* and *cim5-1* cells under the Single and Multi conditions (**F**).

Given that dosage compensation is performed by post-transcriptional mechanisms (Fig 1D), the deceleration of protein synthesis and/or the acceleration of protein degradation should be the mechanisms of dosage compensation (S6 Fig). We first examined the contribution of protein degradation by focusing on the ubiquitin–proteasome system, a major selective degradation pathway. We used *cim5-1* strain as a proteasome-defective mutant [23] to test whether the compensation is not observed in this mutant. As shown in Fig 2A and 2B, the dosage compensations of Rbg1, Mtw1, and Erp2 were significantly weaker in *cim5-1* than in wild-type cells (*CIM5*). The compensations of Pop5 and Saw1 also tended to be weaker in *cim5-1* mutant, although the difference was not statistically significant (S7 Fig). The mRNA levels of these genes in *cim5-1* and *CIM5* cells did not differ (S8 Fig).

To further verify the participation of the ubiquitin–proteasome system in dosage compensation, we examined the ubiquitination of the compensated proteins. The TAP-tagged proteins were immunoprecipitated with IgG-coated beads and cleaved with tobacco etch virus (TEV) protease, and the cleaved proteins were analyzed by Western blotting using anti-ubiquitin antibody (Fig 2C). Because the expression levels of the dosage-compensated proteins and the pull-down efficiency were different among the samples (Fig 2D), we normalized the ubiquitination level by dividing it by loading amount of immunoprecipitated proteins as described in Fig 2E. We compared the amount of the TAP-tagged proteins captured on the beads before and after TEV cleavage, which reflects the amount of immunoprecipitates analyzed by Western blotting for ubiquitinated proteins. This analysis showed a tendency to accumulate the greater amount of ubiquitinated proteins in *cim5-1* cells upon the Multi condition (Fig 2F). These results strongly suggest that protein degradation by the ubiquitin–proteasome system is the main mechanism of dosage compensation.

### Translational efficiency is not changed during dosage compensation of *POP5*

**Fig 3:**
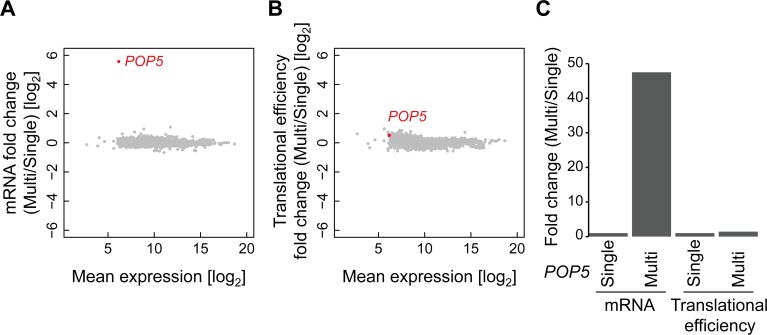
Translational efficiency of *POP5* is not changed during dosage compensation. (**A, B**) Scatter plots showing the changes in mRNA levels (**A**) and the translational efficiency (**B**) of the genome in the Pop5-TAP strain carrying multicopy plasmid pTOWug2-*POP5* grown in SC–Ura medium. The X-axes indicate the mRNA level of each gene obtained by RNA-seq (mean counts in RNA-seq). The translational efficiency of each gene was calculated by dividing the ribosome density by the mRNA level. The mean fold changes relative to the Single condition were calculated from two biological replicates. (**C**) Bar graph indicates the mRNA level and translational efficiency of *POP5* shown in Fig 3A and 3B.

We also examined the contribution of translational control to dosage compensation. A high compensation level of Pop5 in *cim5-1* cells (Fig 2B) prompted us to measure the translational efficiency change upon an increase in *POP5* copy number. We performed ribosome profiling and RNA-seq and measured translation rate comparing between the Single and Multi conditions of *POP5* gene copy number. While a high copy number of *POP5* led to an increase in its mRNA expression (Fig 3A and 3C), the ribosome density per mRNA was not changed (Fig 3B and 3C). The RNA-seq analysis also indicates that an increase in *POP5* copy number by the gTOW technique specifically increased its mRNA level and did not induce off-target effects on mRNA expression of the other genes. Therefore, we conclude that translational efficiency is not responsible for dosage compensation, at least in the case of Pop5. Residual proteasome activity in *cim5-1* mutant or alternative systems may specifically degrade Pop5 protein upon an increase in its gene copy number.

### Complex subunits are predominant target of dosage compensation

**Fig 4:**
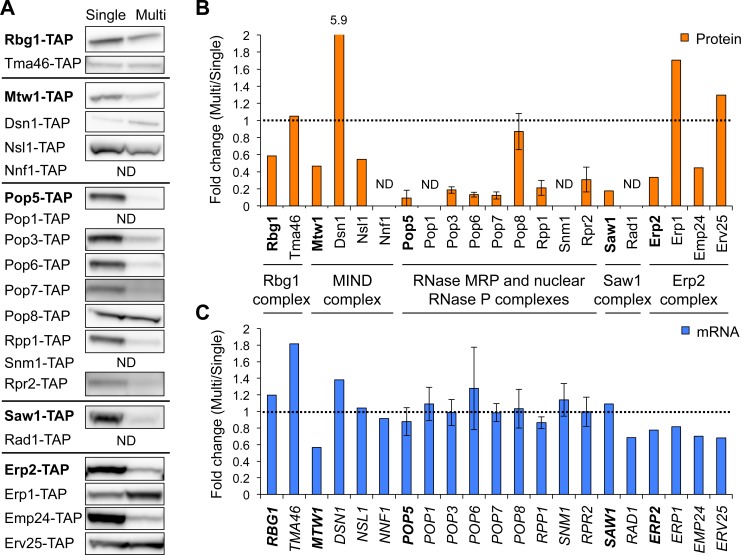
Complex subunits tend to be subjected to dosage compensation. (**A**) Western blots of subunits composed of the five complexes. The experiments were performed using the same method with the screening. TAP-tagged target proteins expressed from the genomic regions were detected with PAP. The dosage-compensated proteins identified from the screening are shown in bold letters. (**B**) Quantification of protein expressions of the subunit genes. (**C**) Quantification of mRNA expressions of the subunit genes. The mRNA level of each TAP-tagged target gene was measured as described above. Dashed line denotes the same expression level between the Multi and Single conditions. The RNase MRP and nuclear RNase P subunit genes were analyzed in three biological replicates, and the average fold changes ± s.d. were calculated relative to the Single condition. ND: not detected.

We noted that all the five dosage-compensated genes identified by the chromosome I screen encode subunits of protein complexes, as listed in Table 1. To investigate the relationship between dosage compensation and complex subunits, we analyzed other subunits of the complexes. As shown in Fig 4, we found that six of seven subunits of the RNase MRP and nuclear RNase P complexes, *NSL1* in the MIND complex, and *EMP24* in the Erp2 complex were compensated at the protein level but not at the mRNA level. Quantification showed that the degree of compensation is very similar among the six subunits of the RNase MRP and nuclear RNase P complexes (Fig 4B). As listed in Table 1, we tested an additional 12 subunit genes and identified 7 dosage-compensated ones. This ratio is significantly higher than that identified in the initial screening (5 out of 54 genes) (*p* < 10^−9^, chi-square test), although not all subunit genes are compensated. Thus, we speculated that dosage compensation predominantly targets complex subunits.

**Table 1:** Relationship between dosage compensation and complex subunits.

Gene	Complex	Subunit*^a^*	Tested subunit*^b^*	Compensated subunit	Reference
*RBG1*	Rbg1–Tma46	2	2	1	[24]
*MTW1*	Mtw1–Nnf1–Nsl1–Dsn1 (MIND)	4	3	2	[25]
*POP5*	RNase MRP and nuclear RNase P	10	7	6	[26]
*SAW1*	Saw1–Rad1–Rad10	3	1	1	[27]
*ERP2*	Erp2–Erp1–Emp24–Erv25	4	4	2	[28]

^*a*^ This number includes only protein subunits, not RNA subunits.

^*b*^ This number does not include subunits whose protein expression is not detected.

### Dosage compensation affects not only subunit levels but also complex levels

**Fig 5:**
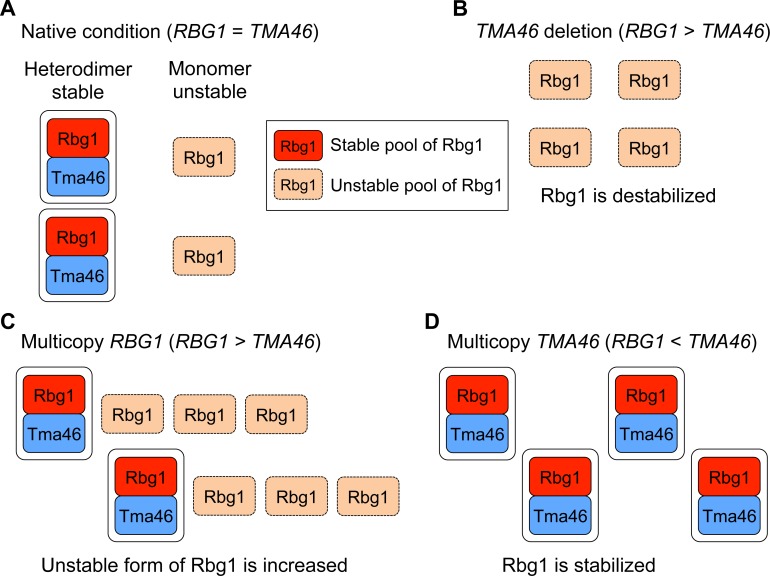
Model for maintaining subunit stoichiometry in the Rbg1–Tma46 heterodimer. (**A**) Under native conditions, monomeric Rbg1 becomes more stable when it forms a complex with Tma46. (**B**) Upon deletion of *TMA46*, Rbg1 is destabilized due to a loss of the partner subunit and rapidly degraded. The unstable pool of Rbg1 is predominant in this condition. (**C**) Upon multicopy expression of Rbg1, the level of the unstable form of Rbg1 is increased, which is targeted for rapid degradation. The unstable pool of Rbg1 is predominant in this condition. (**D**) Upon multicopy expression of Tma46, potentially degraded Rbg1 is stabilized by forming the Rbg1–Tma46 complex with an excess of Tma46. The stable pool of Rbg1 is predominant in this condition.

**Fig 6:**
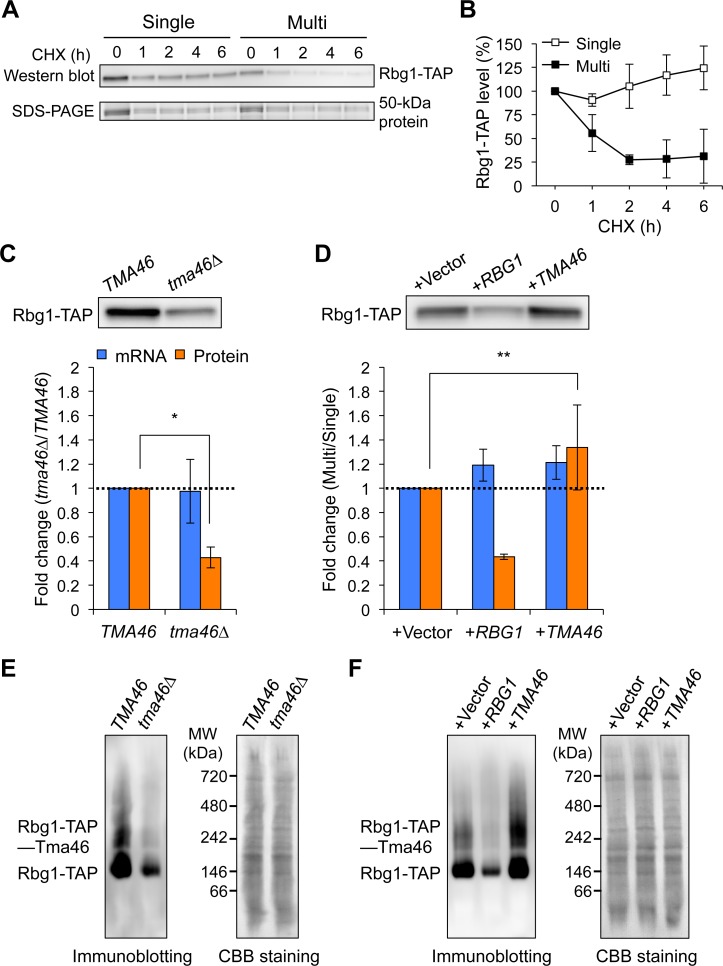
Dosage compensation through accelerated proteolysis buffers genetic perturbations to subunit stoichiometries at the complex level. (**A**) Rbg1-TAP expressed in the Single and Multi conditions was detected by Western blotting with PAP after CHX treatment. A 50-kDa protein, corresponding to enolase, is used as a loading control. (**B**) Quantification of the degradation of Rbg1-TAP. Percentage of the Rbg1-TAP level at each time point relative to the time point 0 is shown. The average expression levels ± s.d. were calculated from three biological replicates. (**C**) The effect of *TMA46* deletion on Rbg1 expression. Rbg1-TAP expressed in wild-type and *tma46Δ* cells grown in YPD medium was detected by Western blotting with PAP (upper panel), and the fold changes were calculated (lower panel). The mRNA levels were measured as described above. The average fold changes ± s.d. from three biological replicates were calculated relative to the wild-type strain. (**D**) The effect of multicopy *TMA46* on Rbg1 expression. Rbg1-TAP expressed under the Single (+Vector) and Multi (+*RBG1*: multicopy of *RBG1*, +*TMA46*: multicopy of *TMA46*) conditions were detected by Western blotting with PAP (upper panel), and the fold changes were calculated (lower panel). The mRNA levels were measured as described above. These cells were grown in SC–Leu–Ura to increase the plasmid copy number. The average fold changes ± s.d. of mRNA and protein levels relative to the Single condition were calculated from three and six biological replicates, respectively. Dashed line denotes the same expression level between the Multi and Single conditions. Statistical significance was determined by a one-tailed Mann Whitney *U* test (*P = 0.05, **P < 0.03). (**E, F**) The effect of *TMA46* deletion (**E**) and multicopy of *RBG1* or *TMA46* (**F**) on the levels of the Rbg1–Tma46 complex. The wild-type and *tma46Δ* cells without the plasmids and wild-type cells with the plasmids were grown in YPD and SC–Ura media, respectively. Rbg1-TAP and Rbg1-TAP–Tma46 expressed in these cells were detected by Native-PAGE followed by immunoblotting with PAP. Left: immunoblotting with PAP. Right: total protein blotted onto the membrane and stained with Coomassie brilliant blue (CBB) R250. A representative blot from two biological replicates is shown.

As shown above, dosage compensation may be performed mainly through protein degradation and target predominantly complex subunits. We thus hypothesized that accelerated degradation of excess subunits that failed to construct a stable complex is the nature of dosage compensation. To examine this, we focused on the Rbg1–Tma46 complex as a model complex. Our working hypothesis is that when a subunit is overexpressed, there are two pools of subunit, the unstable pool that has not found a dimerization partner and the stable pool that is in a complex (Fig 5). The unstable pool is present but very small in the native condition where a large fraction of Rbg1 molecules are stable and a stoichiometric balance between Rbg1 and Tma46 is in the steady state. In contrast, when Rbg1 is overexpressed, the unstable pool of Rbg1 is predominant. In the unstable pool, accelerated degradation of excess subunits should be observed. We first assessed the degradation of Rbg1 upon its overexpression by measuring the amount of Rbg1 after treating cells with a translational inhibitor, cycloheximide (CHX). The CHX chase assay showed accelerated degradation of Rbg1 when its gene copy number was increased (Fig 6A and 6B), as we expected.

We next tested the effect of a loss and a high copy number of *TMA46* on Rbg1 expression. In *tma46Δ* strain, the Rbg1 expression was reduced to less than 0.5-fold (Fig 6C and S9A Fig). On the other hand, the amount of Rbg1 was increased more than 1.3-fold when the *TMA46* copy number was increased in wild-type cells (Fig 6D and S9B Fig). These compensations are performed post-transcriptionally because the *RBG1* mRNA levels were not changed in these conditions (Fig 6C and 6D). We further examined whether dosage compensation directly contributes to a higher or lower levels of the resulting complexes. The levels of the Rbg1–Tma46 complex were assessed by Native-PAGE followed by immunoblotting. This analysis confirmed that the complex was almost not detected in *tma46Δ* strain (Fig 6E). In wild-type cells, the levels of the TAP-tagged version of the Rbg1–Tma46 complex decreased and increased upon an increase in *RBG1* and *TMA46* copy numbers, respectively (Fig 6F). These changes in the complex levels are consistent with the changes in the Rbg1 monomer levels in the same conditions. Therefore, we conclude that Rbg1 stability is modulated depending on the dosage balance against the partner molecule Tma46 and that dosage compensation affects not only subunit levels but also complex levels.

## Discussion

This study extends our understanding of the rescue mechanism for perturbations causing the breakdown of biological systems. Our results demonstrate that protein-level dosage compensation is responsible for robust expression of subunit genes under genetic perturbations. Correction of the subunit levels is performed at the final step in gene expression by protein degradation rather than earlier steps, mRNA transcription/degradation or translation. These results suggest that dosage compensation at the post-translational level is a critical step to mask the fragility caused by an increase in gene copy number. Furthermore, our findings in the context of systems biology provide a new foundation for the robustness of cellular systems.

The robustness in cellular systems to gene copy number changes has been investigated mainly using two approaches: generating aneuploidy of specific chromosomes [12,18] and introducing a plasmid carrying an individual target gene [17]. The generation of aneuploid cells containing one extra chromosome doubles the number of genes in the additional chromosome. Several recent studies using aneuploid yeast and mammalian cells have revealed fragility of cellular systems against gene copy number increase in a genome-wide manner [12,18]. The use of a multicopy plasmid carrying an individual target gene dramatically increases its copy number. A particular method for this approach is based on the gTOW technique [17]. The genome-wide gTOW analysis has revealed over 80% of the yeast genome with more than 100 copies of an upper limit of gene copy number.

The impact of an increase in gene copy number on cell fitness differs between doubled number of genes in an extra chromosome and many copies of a single gene. Previous studies have demonstrated that aneuploidy-induced proteotoxic stress causes cell fragility leading to growth impairment [10,13,29]. Because aneuploid yeast strains are very sensitive to perturbations at the RNA and protein levels, aneuploidy-induced proteotoxicity affects a wide range of biological processes. On the other hand, overexpression of most individual genes does not inhibit growth of wild-type yeast strain [17,30]. Thus, the gTOW technique allows us to study mechanisms for buffering against genetic perturbations by focusing on individual target genes in normal physiological condition. We expect that exploring the effects of an increase in individual gene copy number will identify novel mechanisms for maintaining cellular homeostasis. Indeed, a very recent study has shown that the fragility of aneuploid cells is caused by many genes on single additional chromosomes but not by duplicated dosage-sensitive genes that were identified by the gTOW analysis [31].

We first developed a screening method based on the gTOW technique to estimate how much of the genome is subjected to dosage compensation (Fig 1A). Our screen of chromosome I showed that 5 out of 54 genes are regulated by the compensation (Fig 1B), which estimates that dosage compensation confers robustness to 10% of the genome for buffering perturbed gene expression. Interestingly, all screened genes encode subunits of different complexes (Table 1) and, for 17 subunits included in these complexes, 70% (12 subunits) are subjected to dosage compensation (Fig 4 and Table 1). This result is in agreement with previous findings that protein levels of duplicated genes encoding complex subunits are reduced in aneuploid yeast strains [10]. However, Mtw1 and Rpp1, the dosage-compensated proteins identified in this study, are not compensated in aneuploid cells [12]. This difference may result from aneuploidy-specific physiological conditions associated with proteotoxicity [32].

Given that the biological function of dosage compensation is to maintain subunit stoichiometry, this result explains our previous observation that cellular systems are very fragile to subunit gene overexpression [17]. This is also consistent with previous observations that the stoichiometric imbalance caused by aneuploidy strongly correlates with impaired cell growth [18,33]. Similarly, our data support a classical hypothesis called the balance hypothesis that predicts deleterious effects due to imbalanced subunit stoichiometry [34].

Recent studies investigating the robust formation of protein complexes have elucidated the location where subunits are translated [35,36], the timing when subunits are assembled into complexes [37], and the mechanisms by which subunit stoichiometry is maintained [38,39]. Li et al. found a proportional synthesis strategy whereby protein synthesis rates of complex subunits correlate with subunit stoichiometry [39]. This strategy guarantees stoichiometry of some well-characterized complexes, with a small number of exceptions synthesized in excess. In agreement with previous studies [29,38,40–43], we also identify proteasomal degradation as a mechanism of dosage compensation. We further provide direct evidence for the ubiquitination of the individual dosage-compensated proteins (Fig 2C). Thus, this study enhances our understanding of dosage compensation as a general mechanism for the fine-tuning of subunit levels.

Protein-level dosage compensation might occur cotranslationally for the following reasons: (i) Subunits are assembled into complexes cotranslationally [37]. (ii) A large proportion of the proteome is cotranslationally ubiquitinated [44,45]. (iii) The degradation of subunits via an N-terminal degradation signal at the nascent chain level has been supported by experimental evidence [38]. In addition, autophagy might be included because higher expression of autophagy-related proteins has been detected in aneuploid mammalian cells [15,18].

We show no evidence for a contribution of translational efficiency to the compensation of Pop5 protein (Fig 3B and 3C). This result supports the robust translational efficiency of duplicated genes in aneuploid yeast strains [12,33]. However, it should be noted that an increase in a single gene to approximately 20 copies does not result in a decrease in ribosome occupancy for its mRNAs (S3 Fig, Fig 3B and 3C). We speculate that translational efficiency is not responsible for dosage compensation and that translation is quite robust against genetic perturbations caused by an increase in gene copy number.

Although our screen of chromosome I suggests that the dosage-compensated genes encoding complex subunits constitute approximately 10% of the genome, subunit genes constitute 33% of the yeast genome. This suggests that there are other rules to distinguish between the compensated subunits and the uncompensated ones. Pop8 might be helpful for further characterization of the dosage compensation mechanism since the compensation level of only Pop8 differed from those of all other tested subunits of RNase MRP and nuclear RNase P complexes (Fig 4). Pop8 has the smallest number of interacting partners in these complexes, although the other subunits have at least two or more potential partners [26,46]. Therefore, Pop8 is suggested to be located at the peripheral region of these complexes. It is also known that only depletion of the Pop8 does not result in deleterious effects on RNase MRP function [46–51]. A similar observation in a different protein complex, oligosaccharyl transferase (OST), was recently reported [42]. The OST complex consists of nine subunits, including the functionally redundant Ost3 or Ost6 components, which are potentially the last subunit assembled into the complex. Overexpression of Ost3 or Ost6 does not lead to reduction of its protein level, whereas many of the other subunits show accelerated degradation upon their overexpression. Moreover, deletion of the Ost3 or Ost6 gene does not affect the protein level of the other subunits and results in only a small decrease in enzyme activity of the OST complex [42,52,53]. As listed above, characteristic features with similarities between Pop8 and Ost3 or Ost6 include the order of assembly, number of interactions, and responsibility for the function of each complex. Consideration of these features seems to provide other rules to determine the complex subunits predominantly regulated by dosage compensation.

As shown in Fig 6, we show that the compensation of Rbg1 is performed in a stoichiometry-dependent manner between gene dosage of *RBG1* and *TMA46*. This bidirectional regulation of Rbg1 level may reflect changes in its degradation rate (Fig 6A and 6B). These results are analogous to bidirectional changes of Cog1 level upon overexpression of itself or its partner subunits: Cog2, Cog3, and Cog4 [38]. Although dosage compensation has been postulated to contribute to the levels of subunits and also resulting complexes, there might be no direct evidence for changes in the complex levels. Our study provides direct experimental evidence that dosage compensation of Rbg1 affects the levels of the Rbg1–Tma46 complex under genetic perturbations (Fig 6E and 6F).

We conclude by noting that subunit stoichiometry potentially has a broad impact on robustness in cellular systems because of the fact that numerous biological processes are dependent on protein complexes. Furthermore, studies of mechanisms behind stoichiometry maintenance might be important for understanding diseases related to gene copy number alterations. For example, a recent study suggests that a set of specific genes on trisomic chromosome 21 have a causal effect on Down syndrome [54]. Again, our approach based on the gTOW technique for measuring robustness in cellular systems provides a fundamental framework for the quantitative assessment of cell robustness.

## Materials and Methods

### Strains, plasmids, and media

The yeast strain BY4741 (*MATa his3Δ1 leu2Δ0 met15Δ0 ura3Δ0*) [55] was used for the screening, ribosome profiling, and protein complex analysis. The W303-1B (*MATα ade2-1 his3-11*,*15 leu2-3*,*112 trp1-1 ura3-1 can1-100*) [56] and CMY765 (*MATα cim5-1 ura3-52 leu2Δ1 his3Δ200*) [23] strains were used for the analysis of the ubiquitin–proteasome system. The *tma46Δ* strain (*MATa tma46Δ*::*KanMX his3Δ1 leu2Δ0 met15Δ0 ura3Δ0*) was also used for the protein complex analysis. TAP-tagged or GFP-tagged strains (BY4741 background) and *tma46Δ* strain were obtained from Thermo Scientific. These strains were transformed with empty vector pTOWug2-836 or pTOW40836 or the same vector carrying the gene of interest. Transformation of the yeast strains was performed by the lithium acetate method [57]. The transformants were grown at 30°C in SC medium lacking the indicated amino acids.

### Measurement of gene copy number

The copy number of each gene was measured using the gTOW technique, as described previously [17]. Briefly, single colonies of yeast cells carrying pTOW plasmids were cultivated in a 96-well plate containing 200 μL of SC–Ura medium for 4 days at 30°C, and then, 5 μL of the culture was inoculated into 200 μL of fresh SC–Ura medium. After culturing for 50 h at 30°C, the cells were harvested by filtration followed by DNA extraction with zymolyase treatment. The extracts were subjected to real-time quantitative PCR with Lightcycler 480 (Roche) using SYBR Green I Master (Roche) to quantify the expression of *LEU3* from the chromosome and *leu2d* gene from pTOW plasmids. The resulting copy number of the pTOW plasmid carrying each target gene was calculated according to the method described previously [19].

### RT-PCR

Yeast cells grown in the appropriate medium were harvested at log-phase and subsequently total RNA was extracted using the hot phenol method [58]. Contaminating genomic DNA was removed and reverse transcription was carried out with PrimeScript RT reagent Kit with gDNA Eraser (TaKaRa) according to the manufacturer’s instructions. The generated cDNA was amplified by real-time quantitative PCR with Lightcycler 480 using SYBR Green I Master. Quantification of *TAP* tag and *ACT1* mRNA expression was performed with the following primers to amplify *TAP*-tag and *ACT1* gene on the chromosome: *TAP*-tag-forward (5´-AATTTCATAGCCGTCTCAGCA-3´); *TAP*-tag-reverse (5´-CTCGCTAGCAGTAGTTGGAATATCA-3´); *ACT1*-forward (5´-TGCAAACCGCTGCTCAA-3´); and *ACT1*-reverse (5´-TCCTTACGGACATCGACATCA-3´). The fold change of mRNA levels was calculated as previously described [11].

### Western blot analysis

Yeast cells were grown in 2 mL of the appropriate medium and subcultured in 3 mL of fresh medium. The optical density at 600 nm (OD_600_) was measured and 2 OD_600_ units were harvested at log-phase. The cells were treated with 1 mL of 0.2 N NaOH for 5 min at room temperature and then were suspended in 2× NuPAGE LDS Sample Buffer (Invitrogen) and heated at 70°C for 10 min. The supernatant corresponding to 0.5 OD_600_ units was labeled with EzLabel FluoroNeo (ATTO) and subjected to polyacrylamide gel electrophoresis with lithium dodecyl sulfate (SDS-PAGE), followed by Western blotting with PAP (Sigma-Aldrich) (1:2000) or an anti-GFP antibody (Roche) (1:1000) and peroxidase-conjugated secondary antibody (Nichirei Biosciences) (1:1000). We used NuPAGE 4%–12% Bis-Tris Gel (Invitrogen) for SDS-PAGE and iBlot Transfer Stack PVDF membrane (Invitrogen) for Western blotting. Chemiluminescence was induced by SuperSignal West Femto Maximum Sensitivity Substrate (Thermo Scientific) and detected using LAS-4000 image analyzer (Fujifilm) and ImageQuant LAS 4000 (GE Healthcare). The band intensity was quantified using ImageQuant TL (GE Healthcare), and the fold change of protein levels was calculated as shown in S2 Fig according to a previously described method [11].

### TAP pull-down and Western blot analysis of ubiquitinated proteins

TAP-tagged strains carrying pTOW plasmid were cultivated in 100 mL of SC–Ura medium. The whole cells were harvested at log-phase and lysed with glass beads in 750 μL of lysis buffer [20 mM HEPES, 2 mM EDTA, 100 mM NaCl, 20% glycerol, 0.05% IGEPAL CA-630 (Sigma-Aldrich), Protease Inhibitor Cocktail, EDTA-Free (Thermo Scientific)] with 20 mM N-ethylmaleimide. The supernatant was immunoprecipitated using Dynabeads coated with pan-mouse IgG (Life Technologies), as described previously [59]. In short, the supernatant was incubated with 40 μL of Dynabeads in a Thermomixer Comfort (Eppendorf) at 21°C for 2 h with shaking at 1300 rpm. The Dynabeads were washed one time with the lysis buffer and three times with the lysis buffer containing 150 mM NaCl and suspended in 16 μL of AcTEV buffer (Invitrogen) containing 1 mM DTT. Before TEV cleavage, for Western blot analysis of TAP-tagged protein, 2 μL of the suspension was removed and suspended in 10 μL of 2× NuPAGE LDS Sample Buffer and heated at 65°C for 20 min. The remaining Dynabeads were then treated with 1 μL (10 units) of AcTEV protease (Invitrogen) in a Thermomixer Comfort at 4°C for 16 h with shaking at 1300 rpm. The supernatant was subjected to Western blotting with polyclonal rabbit anti-ubiquitin antibody (DAKO) (1:500) as primary antibody and peroxidase-conjugated secondary antibody (Nichirei Biosciences). After TEV cleavage, the Dynabeads were suspended in 14 μL of 2× NuPAGE LDS Sample Buffer and heated at 65°C for 20 min, and 2 μL of the extracts were mixed with 8 μL of 2× NuPAGE LDS Sample Buffer and analyzed by Western blotting with PAP. Detection of chemiluminescence was performed as described above.

### CHX chase experiments

Yeast cells were grown to log-phase in SC–Ura, and 0.5 OD_600_ units were harvested for time point 0. Then, CHX was added to a final concentration of 200 μg/mL. Cells were harvested after 1, 2, 4, and 6 h of CHX treatment, followed by total protein extraction in 2× NuPAGE LDS Sample Buffer. The supernatant corresponding to 0.1 OD_600_ units was analyzed by Western blotting against the TAP tag as described above. The protein level at each time point was calculated as the intensity of Rbg1-TAP from Western blot divided by that of the 50-kDa protein, corresponding to enolase, from SDS-PAGE. The relative level was calculated by dividing the protein level at each time point by that at time point 0.

### Ribosome profiling and RNA-seq

Yeast cells BY4741 expressing *POP5*-*TAP* from a single genomic locus and carrying pTOWug2-836 or pTOWug2-*POP5* were grown in 150 mL of SC–Ura at 30°C with vigorous shaking. These cells were grown from an initial OD_600_ of approximately 0.2 to OD_600_ around 0.7, and the cells were then harvested by vacuum filtration. The cell pellet was immediately immersed in a 50 mL conical tube filled with liquid nitrogen and 2 mL of lysis buffer [10 mM Tris-HCl (pH 7.0), 10 mM Tris-HCl (pH 8.0), 150 mM NaCl, 5 mM MgCl_2_, 1 mM DTT, 1% Triton X-100, 200 μg/mL CHX, 25 U/mL Turbo DNase (Invitrogen)] was dripped into the tube.

Extracts were prepared as previously described [21], except that the frozen cells were pulverized with a mixer mill at 30 Hz. The total amount of RNA in the extracts was quantified using RiboGreen (Invitrogen), and then, 50 μg of total RNA was diluted to 300 μL with the lysis buffer. The sample was subjected to preparation of ribosome footprints according to a previously described method [60]. Briefly, total RNA was treated with RNase I (Epicentre), and then the ribosomal pellet was collected by sucrose cushion centrifugation. RNA was recovered from the pellet with TRIzol (Life Technologies) and purified with Direct-zol RNA MiniPrep (Zymo), followed by isopropanol precipitation. The resulting RNA was subjected to gel electrophoresis, and then, the 26–34-nucleotides regions were excised. The size-selected fragments were subjected to dephosphorylation with T4 PNK (New England Biolabs) and linker ligation with T4 Rnl2 (New England Biolabs). Ribosomal RNA was depleted from the sample using Ribo-Zero Magnetic Gold Kit for yeast (Epicentre). Reverse transcription was carried out with Protoscript II (New England Biolabs) on the rRNA-depleted sample. The reverse transcription product was then separated by gel electrophoresis, and the full-length product was excised.

The size-selected product was circularized with CircLigaseII (Epicentre). The circularized DNA was amplified by 6, 8, 10, 12, and 14 cycles of PCR with Phusion polymerase (New England Biolabs). The PCR products were loaded on gel, and the products of eight cycles were excised. The quality of the PCR product was assessed using Agilent 2200 TapeStation (Agilent Technologies). Deep sequencing (50 bp, single-end reads) was then performed on the Illumina HiSeq 4000 (Illumina). RNA-seq libraries were generated using TruSeq Standard Total RNA Library Prep Kit (Illumina) from total RNA prepared as described above, and then, deep sequencing was performed in the same run with ribosome footprint sequencing.

The profiling analysis was performed according to the method previously described [60,61] with modifications for the analysis of budding yeast profiling. In short, rRNA sequences were aligned to a set of budding yeast rRNA sequences, and then, non-rRNA reads were aligned to the budding yeast transcriptome. A-site offsets of ribosome footprints and mRNA fragments were estimated from 13 to 17 nucleotides for each read length of 26–30 nucleotides and 15 nucleotides for 22–51 nucleotides, respectively. The mapped reads excluding the first 15 codons and last 5 codons were counted based on the A-site offsets. DESeq was used to calculate fold change of RNA expression and translational efficiency [62]. Ribosome profiling and RNA-seq data analysis did not distinguish the reads from endogenous or exogenous *POP5* copies.

### Native-PAGE and immunoblotting

Yeast cells were grown to log-phase in 6 mL of the appropriate medium and 5 OD_600_ units were harvested. The cells were washed with 1 mL of sterile water and lysed with glass beads in 250 μL of Digitonin buffer [1% Digitonin (Invitrogen), 1× NativePAGE Sample Buffer (Invitrogen), Protease Inhibitor Cocktail, EDTA-Free]. The supernatant corresponding to 0.2 OD_600_ units was mixed with NativePAGE 5% G-250 Sample Additive (Invitrogen) (final concentration 0.25%) and loaded on NativePAGE 4–16% Bis-Tris Gel (Invitrogen). The native gel electrophoresis was performed at room temperature with NativePAGE Running Buffer Kit (Invitrogen) according to the manufacturer’s instructions. After electrophoresis, the gel was treated with SDS buffer [1× NuPAGE MOPS SDS Running Buffer (Invitrogen), 1% SDS] for 15 min. The gel was washed five times with 1× NuPAGE MOPS SDS Running Buffer, and then, blotted onto PVDF membrane using the iBlot system. After blotting, the membrane was washed with methanol for 5 min for three times, rinsed with PBST [1× PBS, 0.1% Tween 20] for three times, and washed in PBST for 10 min. The membrane was blocked with 4% skim milk in PBST for 1 h at room temperature before incubation with PAP (1:4000) in the same condition. Chemiluminescence was induced and detected as described above. The membrane was stained with CBB-R250 after immunoblotting.

## Supporting Information

S1 Fig Full results of the screening of the dosage-compensated genes. More than 50% (54 out of 96) of genes on chromosome I were screened. Each rectangle includes the systematic name, standard name, and the result of Western blot (the Multi and Single conditions on the left and right, respectively). Rectangles with a pink line denote the dosage-compensated genes. A chromosome map was adopted and modified from the *Saccharomyces* Genome Database website [63].
(TIF)

S2 Fig The linearity and accuracy of the protein quantification. (**A**) SDS-PAGE of two-fold serially diluted cell lysate. The lysate prepared from cells cultured in the Single or Multi conditions were loaded on the same gel. A red rectangle marks the area of a 50-kDa protein, corresponding to enolase, used as a loading control of Western blot analysis.
(**B**) Western blot of two-fold serially diluted cell lysate. The gel shown in S2A Fig was blotted onto PVDF membrane and Rbg1-TAP in the total lysate was detected by Western blot with PAP. A red rectangle marks the area of Rbg1-TAP.
(**C**) The area of a 50-kDa protein cropped from the gel shown in S2A Fig (upper panel). The signal intensity of each band was measured after background subtraction, and the net intensity was plotted on the y-axis (lower panel). The amount of lysate had a correlation coefficient (*R*^*2*^) of 0.99 with the net intensity in both the Single and Multi conditions.
(**D**) The area of Rbg1-TAP cropped from the membrane shown in S2B Fig (upper panel). The net intensity of each band was measured after background subtraction, and the net intensity was plotted on the y-axis (lower panel). The amount of lysate had a correlation with the net intensity of Rbg1-TAP in the Single and Multi conditions (*R*^*2*^ = 0.97 and 0.99, respectively).
(**E**) Quantification of fold change in Rbg1-TAP level between the Single and Multi conditions. The case of analyzing 0.6 OD_600_ units of cells is shown as an example. The net intensities of a 50-kDa protein and Rbg1-TAP from the Multi condition were divided by those from the Single condition to calculate the PAGE fold change and the WB fold change, respectively. Protein fold change was calculated by dividing the WB fold change by the PAGE fold change.
(**F**) The protein fold change calculated from the analysis of each OD_600_ units of cells. Only non-saturated signals were used for all the quantification analysis. Dashed line denotes the same expression level between the Multi and Single conditions. For comparison, the result of the Western blot analysis using 0.5 OD_600_ units of cells, the same data shown in Fig 1C, is shown.
(TIF)

S3 Fig Gene copy number during dosage compensation. Bar graph indicates the copy numbers of pTOWug2-836 (Vector) and the plasmid carrying each of the indicated genes in each TAP-tagged strain. The copy numbers were measured by the gTOW technique. The average copy numbers ± s.d. were calculated from four biological replicates.
(TIF)

S4 Fig Observation of dosage compensation in the analysis of endogenous and exogenous protein levels. (**A**) Schematic overview of the analysis of endogenous and exogenous proteins. Left panel (Single): TAP-tagged strain transformed with the empty vector. The native level of the target protein expressed only from the genomic copy is detected by Western blotting with PAP. Middle and right panels (Multi): TAP-tagged strain transformed with the multicopy plasmid carrying the target gene with the TAP tag. If the level of the TAP-tagged target protein per gene copy is not reduced compared with that in the Single condition (middle panel), the target protein is not subjected to dosage compensation. On the other hand, if the level of the TAP-tagged target protein per gene copy is reduced (right panel), the target protein is subjected to dosage compensation. The cells carrying a multicopy plasmid were grown in SC–Ura medium.
(**B**) Western blot with PAP for the indicated TAP-tagged proteins expressed from the genome and the multicopy plasmid.
(**C**) Quantification of the expression levels of the identified proteins. The average fold changes ± s.d. from three biological replicates were calculated relative to the Single condition. Protein levels at the same dilution in the Multi and Single conditions were used for the quantification.
(**D**) Bar graph indicates the copy number of pTOW40836 carrying each of the indicated genes with the TAP tag. The copy numbers were measured by the gTOW technique. The average copy numbers ± s.d. were calculated from more than three biological replicates.
(**E**) Quantification method of protein fold change per gene copy. The case of analyzing Rbg1 level is shown as an example. The fold change in Rbg1-TAP level between the Single and Multi conditions was divided by the *RBG1* copy number.
(**F**) Bar graph indicates the fold changes of the indicated proteins per gene copy. The average fold changes ± s.d. were calculated from three biological replicates. Dashed line denotes the same expression level between the Multi and Single conditions. For comparison, the result of Western blot analysis detecting the only endogenous target protein, the same data shown in Fig 1C, is shown.
(TIF)

S5 Fig Observation of dosage compensation using the GFP tag. (**A**) Western blot of the dosage-compensated proteins identified from the screen of chromosome I. GFP-tagged target proteins expressed from the genomic regions were detected with an anti-GFP antibody. Pop8 is an example of the uncompensated proteins.
(**B**) Quantification of the expression levels of the indicated proteins. The average fold changes ± s.d. relative to the Single condition were calculated from three biological replicates. Dashed line denotes the same expression level between the Multi and Single conditions. For comparison, the results obtained using the TAP tag, the same data shown in Figs 1C and 4B, are shown.
(TIF)

S6 Fig Possible mechanisms of dosage compensation. (**A, B**) The abundance of mRNA and protein in a cell is a result of a balance between their synthesis and degradation. If a target gene is not subjected to protein-level dosage compensation, an increase in gene copy number results in linear increases in mRNA and protein levels. A deceleration of translation (**A**) and an acceleration of degradation (**B**) can explain a nonlinear relationship between gene copy number and protein level via dosage compensation.
(TIF)

S7 Fig A tendency for reduced levels of dosage compensation in *cim5-1* strain. Bar graph indicates the fold change of each target protein. All data points of Fig 2B are shown. The fold change of Saw1 level in *CIM5* strain of replicate #3 was almost zero.
(TIF)

S8 Fig The mRNA levels of the dosage-compensated genes in *cim5-1* strain. The *TAP* mRNA levels of the indicated genes in *CIM5* (W303-1B) and *cim5-1* (CMY765) strains grown in SC–Ura medium. The mRNA levels were measured by reverse transcriptase-PCR and normalized to *ACT1* mRNA levels. The average fold changes ± s.d. from three biological replicates were calculated relative to the Single condition. Dashed line denotes the same expression level between the Multi and Single conditions.
(TIF)

S9 Fig Bidirectional changes in Rbg1 level in response to *TMA46* copy number. (**A**) The effect of *TMA46* deletion on Rbg1 expression. Rbg1-TAP expressed in wild-type and *tma46Δ* cells grown in YPD medium was detected by Western blotting with PAP, and the fold changes were calculated relative to the wild-type strain. All data points of Fig 6C are shown.
(**B**) The effect of multicopy *TMA46* on Rbg1 expression. Rbg1-TAP expressed under the Single (+Vector) and Multi (+*RBG1*: multicopy of *RBG1*, +*TMA46*: multicopy of *TMA46*) conditions were detected by Western blotting with PAP, and the fold changes were calculated relative to the Single condition. All data points of Fig 6D are shown.
(TIF)
