# Supplementary figures and images for: Post-Translational Dosage Compensation Buffers Genetic Perturbations to Stoichiometry of Protein Complexes
Source: PLoS Genet. 2017 Jan 25;13(1):e1006554. doi: 10.1371/journal.pgen.1006554 (PMC5266272; doi:10.1371/journal.pgen.1006554)

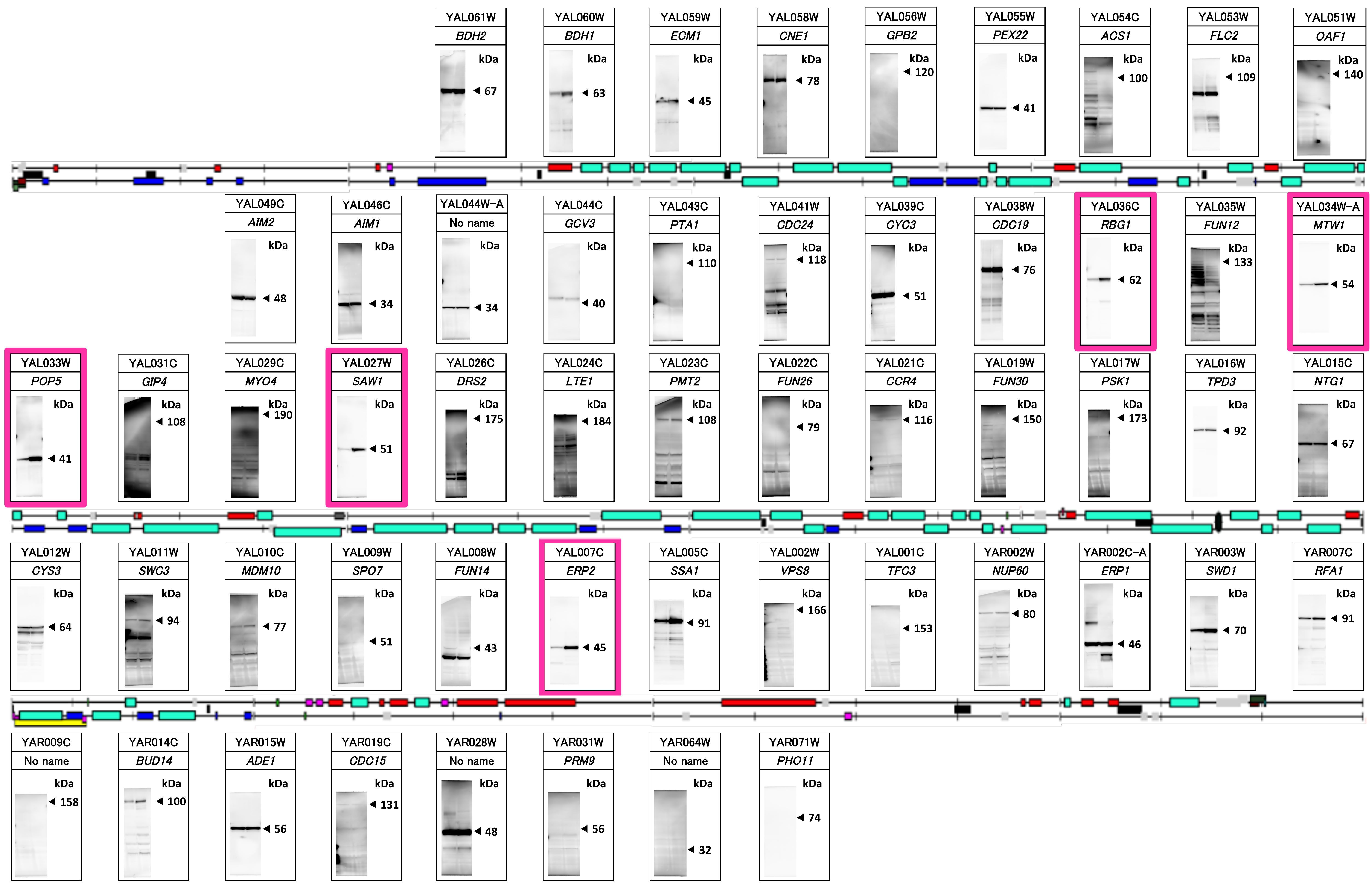

Supplement: S1 Fig — More than 50% (54 out of 96) of genes on chromosome I were screened. Each rectangle includes the systematic name, standard name, and the result of Western blot (the Multi and Single conditions on the left and right, respectively). Rectangles with a pink line denote the dosage-compensated genes. A chromosome map was adopted and modified from the Saccharomyces Genome Database website [63]. (TIF) [file pgen.1006554.s001.tif]

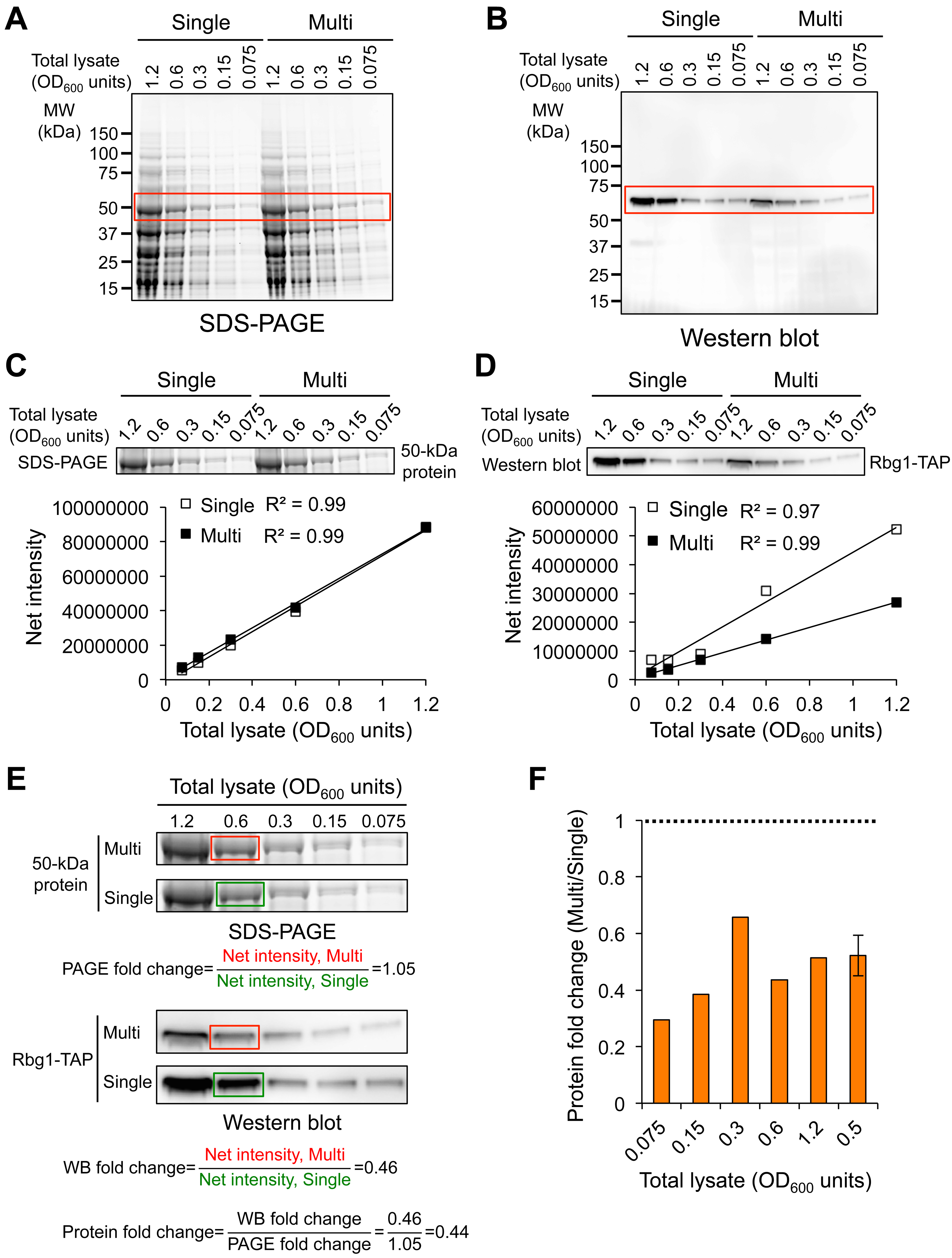

Supplement: S2 Fig — (A) SDS-PAGE of two-fold serially diluted cell lysate. The lysate prepared from cells cultured in the Single or Multi conditions were loaded on the same gel. A red rectangle marks the area of a 50-kDa protein, corresponding to enolase, used as a loading control of Western blot analysis. (B) Western blot of two-fold serially diluted cell lysate. The gel shown in S2A Fig was blotted onto PVDF membrane and Rbg1-TAP in the total lysate was detected by Western blot with PAP. A red rectangle marks the area of Rbg1-TAP. (C) The area of a 50-kDa protein cropped from the gel shown in S2A Fig (upper panel). The signal intensity of each band was measured after background subtraction, and the net intensity was plotted on the y-axis (lower panel). The amount of lysate had a correlation coefficient (R2) of 0.99 with the net intensity in both the Single and Multi conditions. (D) The area of Rbg1-TAP cropped from the membrane shown in S2B Fig (upper panel). The net intensity of each band was measured after background subtraction, and the net intensity was plotted on the y-axis (lower panel). The amount of lysate had a correlation with the net intensity of Rbg1-TAP in the Single and Multi conditions (R2 = 0.97 and 0.99, respectively). (E) Quantification of fold change in Rbg1-TAP level between the Single and Multi conditions. The case of analyzing 0.6 OD600 units of cells is shown as an example. The net intensities of a 50-kDa protein and Rbg1-TAP from the Multi condition were divided by those from the Single condition to calculate the PAGE fold change and the WB fold change, respectively. Protein fold change was calculated by dividing the WB fold change by the PAGE fold change. (F) The protein fold change calculated from the analysis of each OD600 units of cells. Only non-saturated signals were used for all the quantification analysis. Dashed line denotes the same expression level between the Multi and Single conditions. For comparison, the result of the Western blot analysis using [file pgen.1006554.s002.tif]

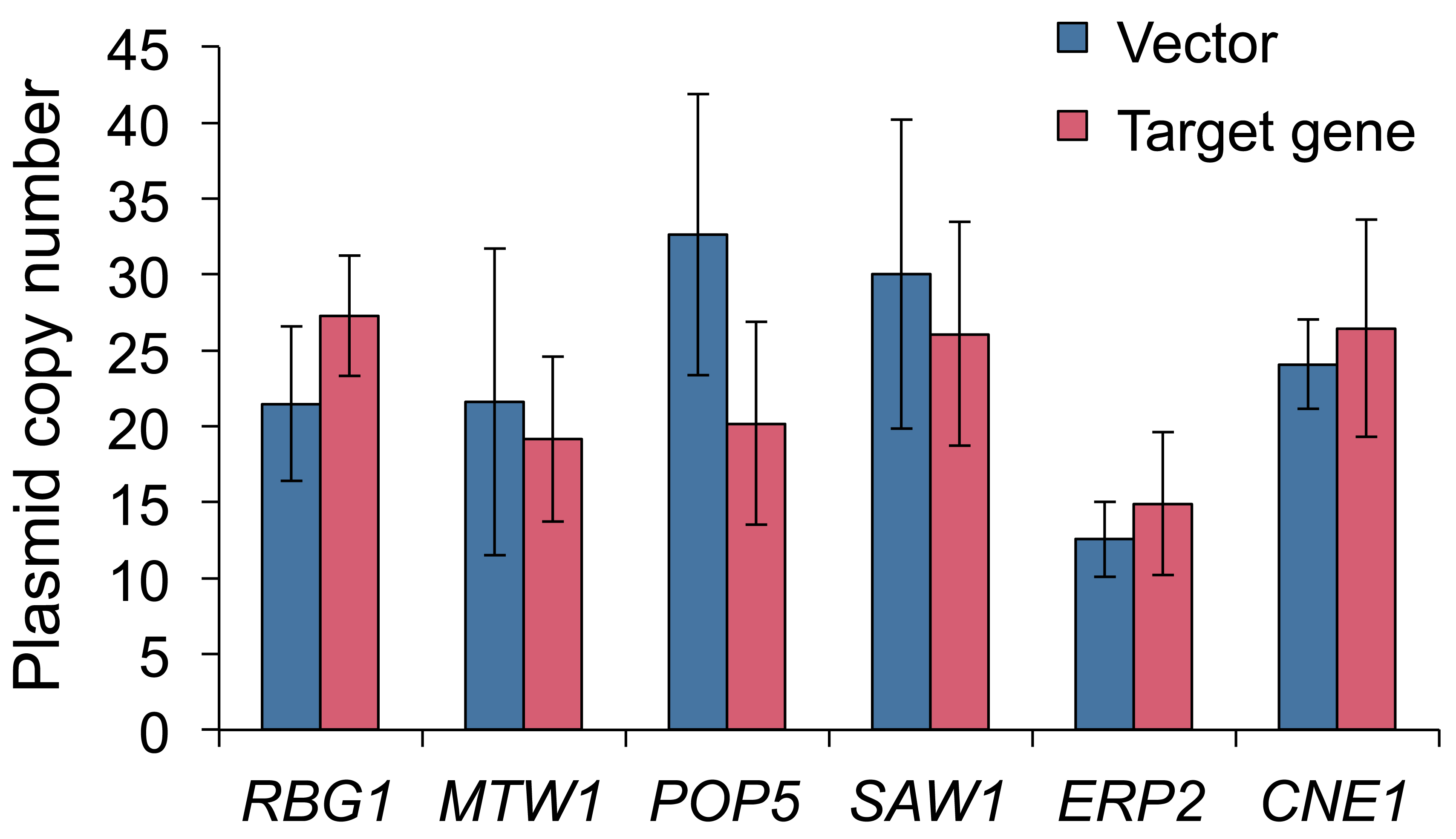

Supplement: S3 Fig — Bar graph indicates the copy numbers of pTOWug2-836 (Vector) and the plasmid carrying each of the indicated genes in each TAP-tagged strain. The copy numbers were measured by the gTOW technique. The average copy numbers ± s.d. were calculated from four biological replicates. (TIF) [file pgen.1006554.s003.tif]

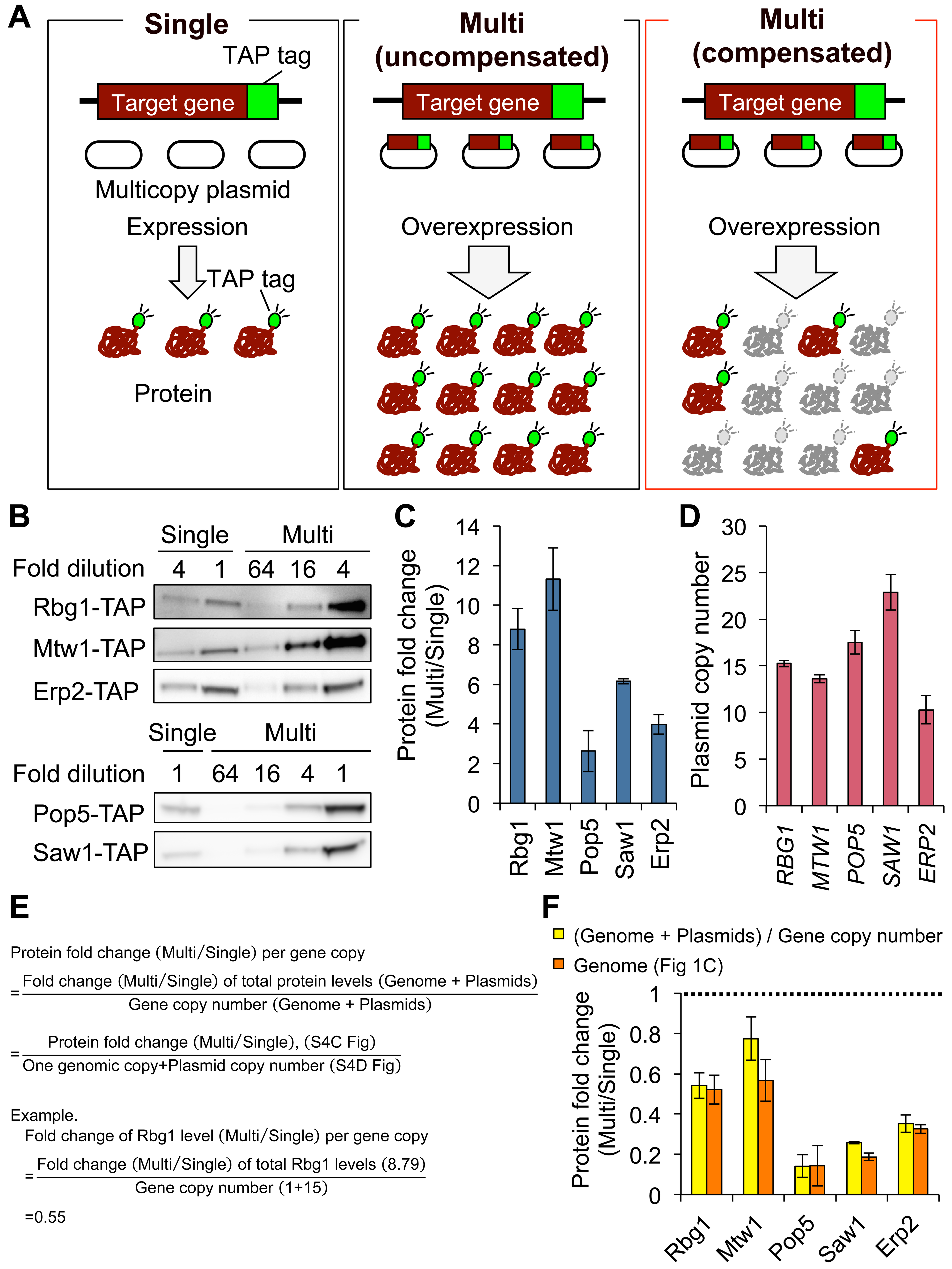

Supplement: S4 Fig — (A) Schematic overview of the analysis of endogenous and exogenous proteins. Left panel (Single): TAP-tagged strain transformed with the empty vector. The native level of the target protein expressed only from the genomic copy is detected by Western blotting with PAP. Middle and right panels (Multi): TAP-tagged strain transformed with the multicopy plasmid carrying the target gene with the TAP tag. If the level of the TAP-tagged target protein per gene copy is not reduced compared with that in the Single condition (middle panel), the target protein is not subjected to dosage compensation. On the other hand, if the level of the TAP-tagged target protein per gene copy is reduced (right panel), the target protein is subjected to dosage compensation. The cells carrying a multicopy plasmid were grown in SC–Ura medium. (B) Western blot with PAP for the indicated TAP-tagged proteins expressed from the genome and the multicopy plasmid. (C) Quantification of the expression levels of the identified proteins. The average fold changes ± s.d. from three biological replicates were calculated relative to the Single condition. Protein levels at the same dilution in the Multi and Single conditions were used for the quantification. (D) Bar graph indicates the copy number of pTOW40836 carrying each of the indicated genes with the TAP tag. The copy numbers were measured by the gTOW technique. The average copy numbers ± s.d. were calculated from more than three biological replicates. (E) Quantification method of protein fold change per gene copy. The case of analyzing Rbg1 level is shown as an example. The fold change in Rbg1-TAP level between the Single and Multi conditions was divided by the RBG1 copy number. (F) Bar graph indicates the fold changes of the indicated proteins per gene copy. The average fold changes ± s.d. were calculated from three biological replicates. Dashed line denotes the same expression level between the Multi and Single conditions. For comparison, the result of [file pgen.1006554.s004.tif]

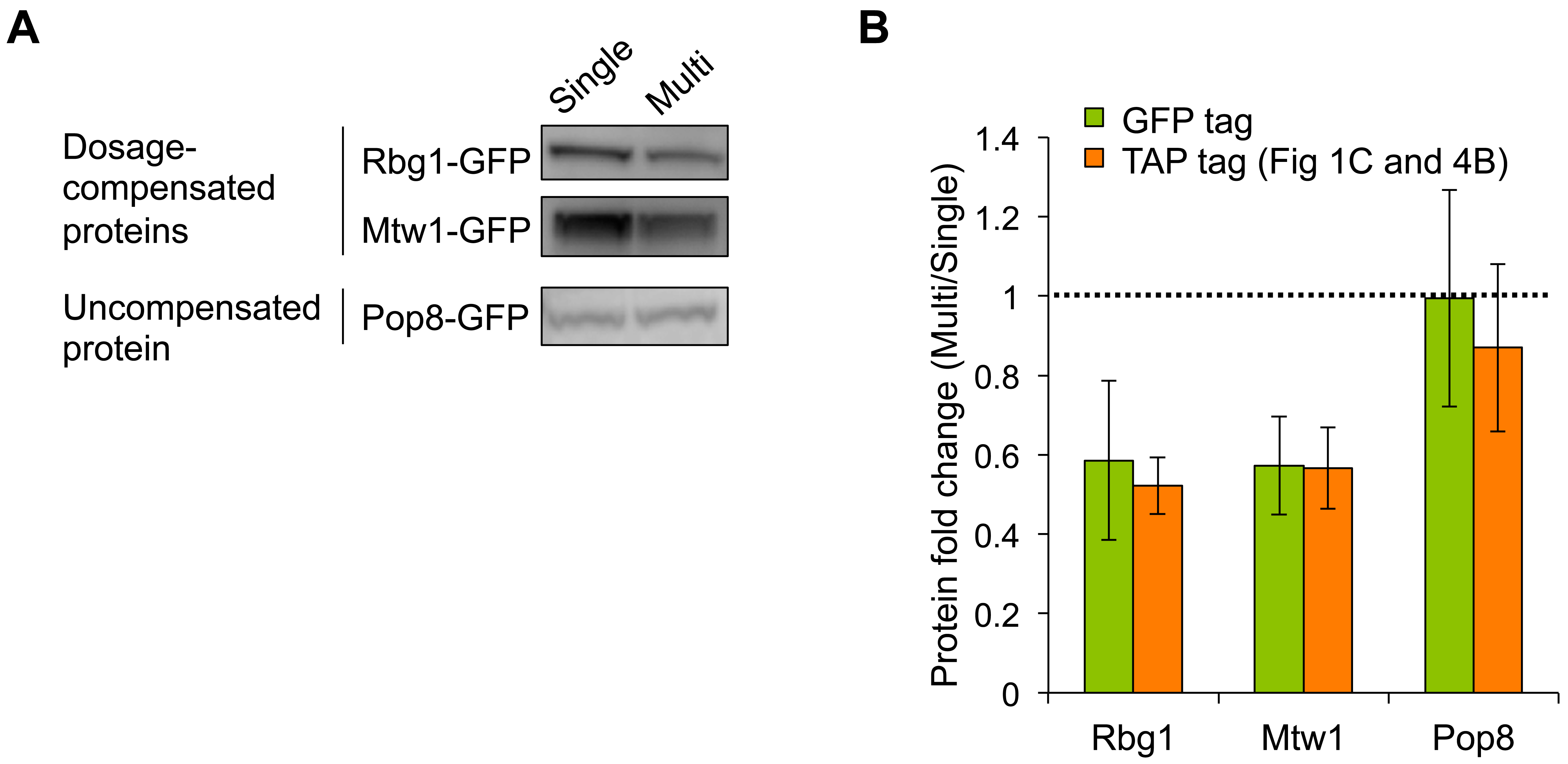

Supplement: S5 Fig — (A) Western blot of the dosage-compensated proteins identified from the screen of chromosome I. GFP-tagged target proteins expressed from the genomic regions were detected with an anti-GFP antibody. Pop8 is an example of the uncompensated proteins. (B) Quantification of the expression levels of the indicated proteins. The average fold changes ± s.d. relative to the Single condition were calculated from three biological replicates. Dashed line denotes the same expression level between the Multi and Single conditions. For comparison, the results obtained using the TAP tag, the same data shown in Figs 1C and 4B, are shown. (TIF) [file pgen.1006554.s005.tif]

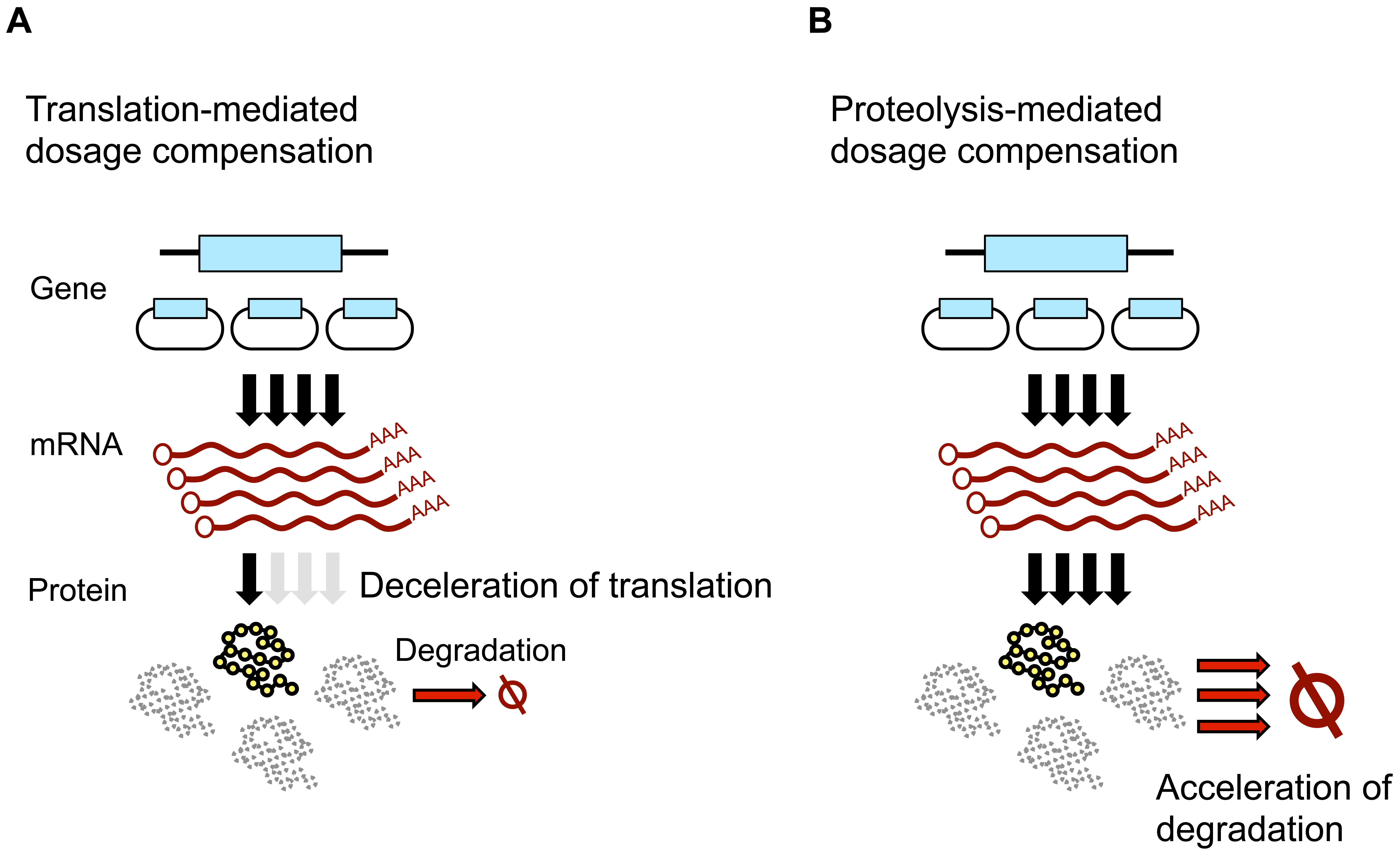

Supplement: S6 Fig — (A, B) The abundance of mRNA and protein in a cell is a result of a balance between their synthesis and degradation. If a target gene is not subjected to protein-level dosage compensation, an increase in gene copy number results in linear increases in mRNA and protein levels. A deceleration of translation (A) and an acceleration of degradation (B) can explain a nonlinear relationship between gene copy number and protein level via dosage compensation. (TIF) [file pgen.1006554.s006.tif]

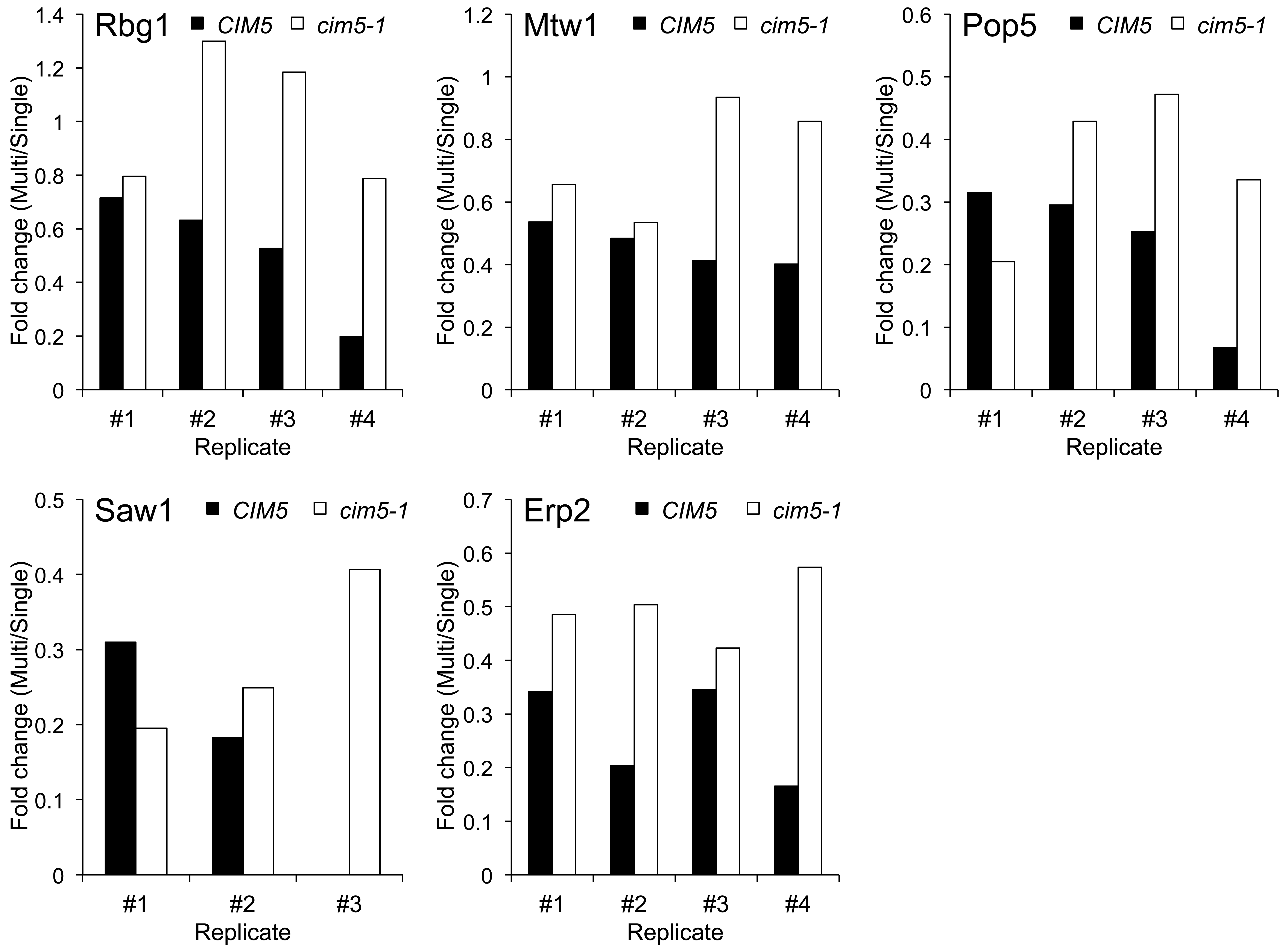

Supplement: S7 Fig — Bar graph indicates the fold change of each target protein. All data points of Fig 2B are shown. The fold change of Saw1 level in CIM5 strain of replicate #3 was almost zero. (TIF) [file pgen.1006554.s007.tif]

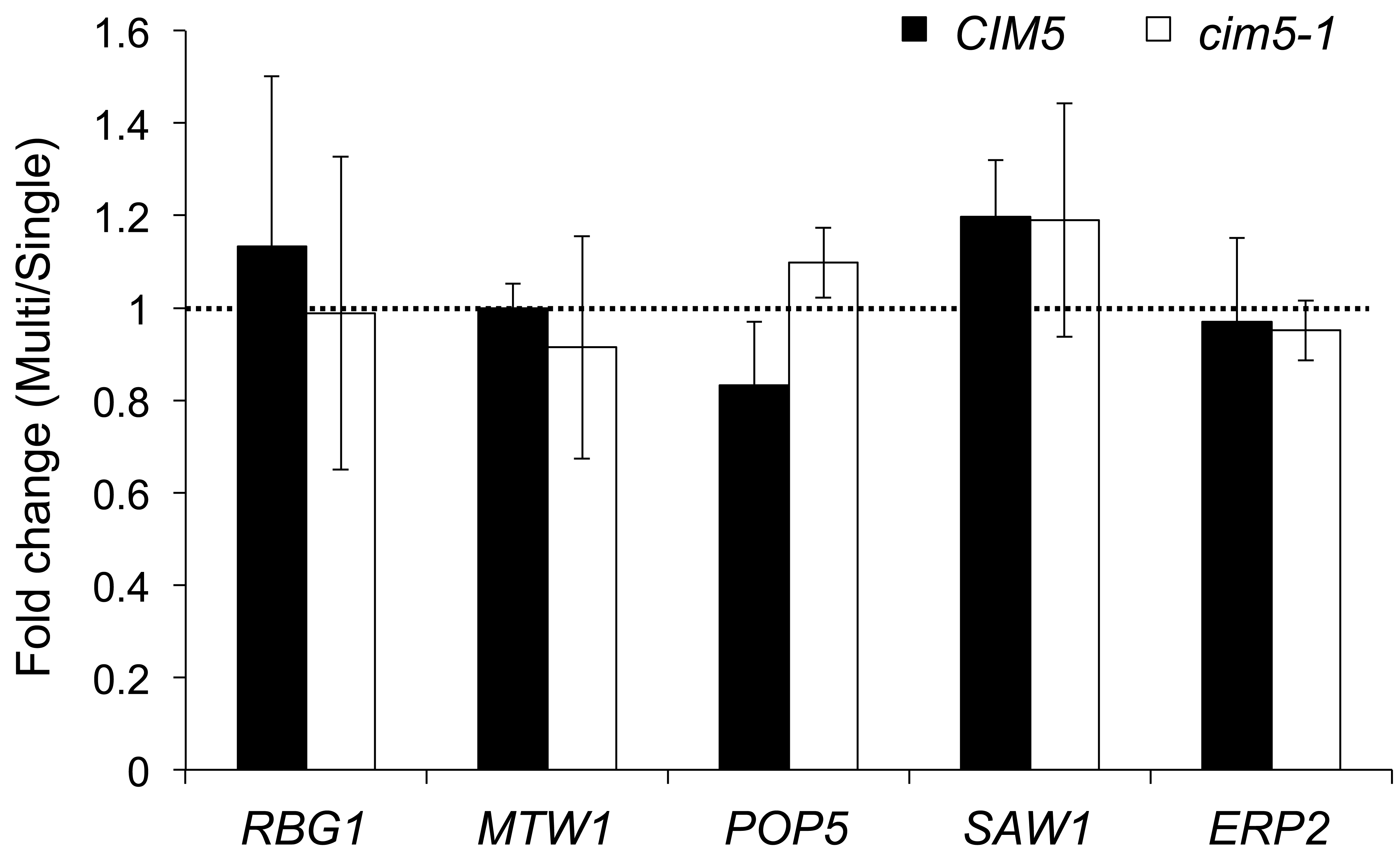

Supplement: S8 Fig — The TAP mRNA levels of the indicated genes in CIM5 (W303-1B) and cim5-1 (CMY765) strains grown in SC–Ura medium. The mRNA levels were measured by reverse transcriptase-PCR and normalized to ACT1 mRNA levels. The average fold changes ± s.d. from three biological replicates were calculated relative to the Single condition. Dashed line denotes the same expression level between the Multi and Single conditions. (TIF) [file pgen.1006554.s008.tif]

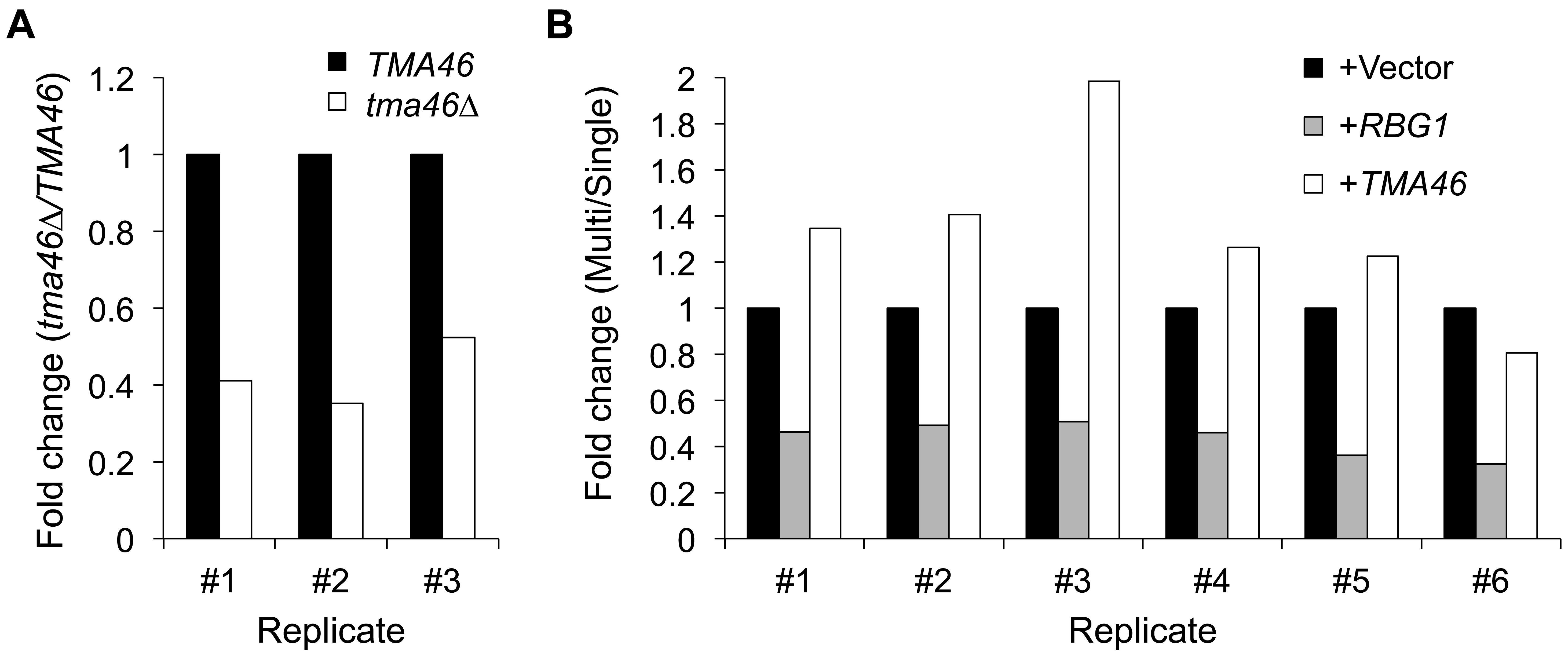

Supplement: S9 Fig — (A) The effect of TMA46 deletion on Rbg1 expression. Rbg1-TAP expressed in wild-type and tma46Δ cells grown in YPD medium was detected by Western blotting with PAP, and the fold changes were calculated relative to the wild-type strain. All data points of Fig 6C are shown. (B) The effect of multicopy TMA46 on Rbg1 expression. Rbg1-TAP expressed under the Single (+Vector) and Multi (+RBG1: multicopy of RBG1, +TMA46: multicopy of TMA46) conditions were detected by Western blotting with PAP, and the fold changes were calculated relative to the Single condition. All data points of Fig 6D are shown. (TIF) [file pgen.1006554.s009.tif]
